# Mini-Review: From measurement to prediction—a conceptual paradigm shift in assessing overcrowding in pediatric emergency departments since 2021

**DOI:** 10.3389/fped.2025.1746637

**Published:** 2026-01-13

**Authors:** Johanna T. Meßner, Melanie L. Conrad, Dennis Freuer, Christine Meisinger, Fabian B. Fahlbusch, Florian Weber

**Affiliations:** 1 Interdisciplinary Pediatric Emergency Department, Faculty of Medicine, University of Augsburg, Augsburg, Germany; 2 Neonatology and Pediatric Intensive Care, Faculty of Medicine, University of Augsburg, Augsburg, Germany; 3 Institute of Microbiology, Infectious Diseases and Immunology, Charité-Universitätsmedizin Berlin, Corporate Member of Freie Universität Berlin, Humboldt-Universität zu Berlin, Berlin, Germany; 4 Epidemiology, Medical Faculty, University of Augsburg, University Hospital Augsburg, Augsburg, Germany

**Keywords:** clinical decision support (CDS) systems, overcrowding assessment, pediatric emergency department (PED), predictive modeling, temporal demand analysis

## Abstract

Overcrowding in pediatric emergency departments (PEDs) is an increasing global challenge. While adult emergency medicine has developed several validated measures to assess overcrowding, pediatric-specific methods remain scarce. In recent years, new approaches have emerged, including the first predictive models capable of anticipating crowding before it occurs. This Mini-Review provides a narrative synthesis of recent conceptual and methodological developments in the measurement and prediction of overcrowding in PEDs, building on the literature published since 2021. It conceptually examines unidimensional metrics, multidimensional scores, and emerging predictive models, emphasizing the shift from retrospective assessment to temporally oriented approaches and the need for pediatric-specific validation and multicenter evaluation.

## 1 Introduction

Overcrowding in emergency departments (EDs) is a growing global challenge that affects healthcare quality, patient safety, and staff well-being (1–6). Pediatric EDs (PEDs) differ substantially from adult settings due to lower admission rates (7–9), higher proportions of non-urgent visits (2, 8, 10, 11), and unique patient flow characteristics (5, 8, 12). These factors limit the direct transferability of adult-derived tools to pediatric contexts.

Reliable measurement of crowding is a prerequisite for effective intervention planning, however, most existing tools such as the National Emergency Department Overcrowding Scale (NEDOCS) or the Emergency Department Work Index (EDWIN) were designed for adult emergency medicine (3, 13). Their use in pediatric settings has only recently been tested, often revealing the need for adaptation. In 2021, the systematic review by Abudan et al. (1) emphasized that no standardized method existed to specifically measure PED overcrowding and called for predictive systems capable of early detection. Since then, two studies have proposed forecasting approaches that anticipate crowding—marking a paradigm shift from reactive detection to proactive management. This Mini-Review maps the development of these approaches from 2021 to 2025 as an updated extension of the synthesis by Abudan et al. (1).

## 2 Methods

**Figure 1:**
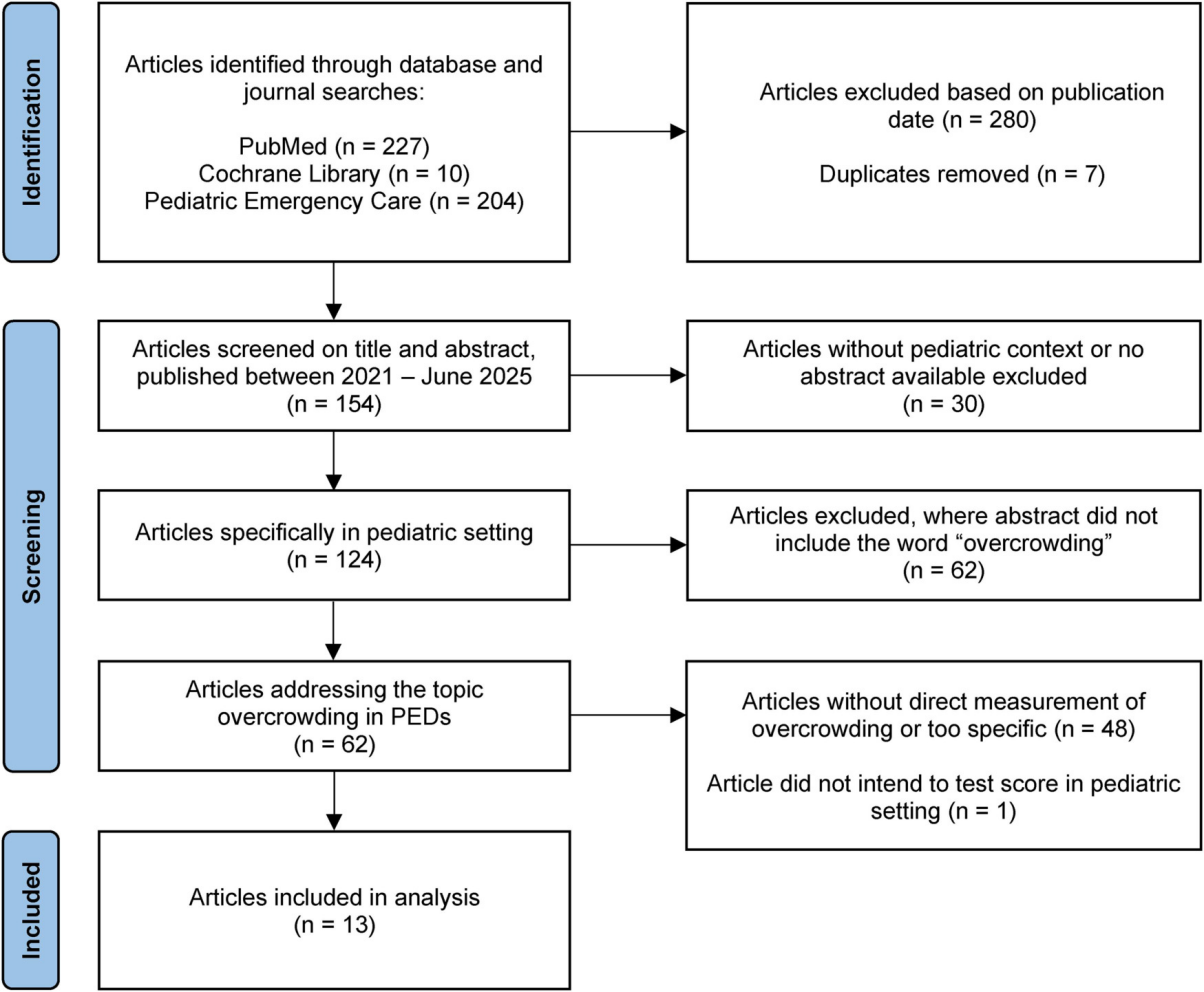
Study selection flow diagram for this narrative Mini-Review. Flow diagram illustrating the identification, screening, and inclusion of studies addressing the measurement or prediction of overcrowding in PEDs since 2021. The diagram is provided to enhance transparency of the literature selection process within the narrative synthesis approach applied in this Mini-Review.

This Mini-Review applies a narrative synthesis approach, informed by a structured literature search to support transparency and conceptual focus. A targeted literature search was conducted in PubMed, the Cochrane Library, and the *Pediatric Emergency Care* journal, covering publications from 2021 to 2025 using the search terms “overcrowding” and “pediatric emergency department.” The selected time window was chosen to capture recent conceptual and methodological developments following the systematic review by Abudan et al. (1). The search strategy was deliberately focused and time-restricted, with the aim of capturing recent conceptual and methodological advances in the assessment and prediction of PED overcrowding. Only peer-reviewed journal articles were considered; gray literature, conference abstracts, and unpublished reports were not included to ensure methodological transparency and consistency of reported metrics. Eligible study designs comprised observational studies, validation studies, and model development and validation studies, including predictive time-series and forecasting approaches. Articles that referred to overcrowding solely as a descriptive or contextual concept without specifying how crowding was operationalized or measured were excluded, as such studies do not allow meaningful comparison of overcrowding assessment or prediction approaches. Duplicates were removed prior to screening, and titles and abstracts were assessed for eligibility according to predefined inclusion criteria. Titles and abstracts were independently reviewed by two reviewers (JTM and FW) to identify publications of conceptual relevance, with full-text articles subsequently assessed for inclusion; any discrepancies were resolved through discussion and consensus. Given the narrative and concept-oriented scope of this Mini-Review, no formal risk-of-bias assessment was performed. Unidimensional metrics and predictive models were distinguished by their temporal orientation, as predictive models enable anticipatory operational planning through forecasts of future crowding—even when based on a single input variable such as patient census—rather than retrospective point-in-time assessment. Pediatric validation status was classified consistently as “pediatric-specific” for tools originally developed for PEDs, “adapted from adult tool” for modified adult-derived scores, and “evaluated in PEDs” for adult tools formally tested in pediatric settings. To enhance transparency of the literature selection process, a study selection flow diagram is provided (Figure 1). The approaches described across the identified publications were conceptually organized into three categories: (1) unidimensional metrics, (2) multidimensional scores, and (3) predictive models, drawing on a total of 13 publications addressing the measurement or prediction of overcrowding in PEDs. The focused and time-restricted nature of the literature search represents an inherent limitation of this Mini-Review. Relevant studies published outside the selected time frame or indexed in additional databases may not have been captured. In addition, restricting inclusion to peer-reviewed journal articles may have excluded emerging approaches reported only in conference proceedings or gray literature.

## 3 Synthesis of recent evidence

Overcrowding assessment tools in pediatric emergency care were grouped into three conceptual categories: unidimensional metrics, multidimensional scores, and predictive models (Table 1). While the first two categories measure crowding retrospectively, the third category represents a move toward predictive management, representing a proactive evolution in managing patient flow and staffing resources.

**Table 1:** Overview of approaches used to assess or predict overcrowding in pediatric emergency departments, conceptually grouped into unidimensional metrics, multidimensional scores, and predictive models.

Tool	Origin	Pediatric validation	Variables vs. reactive	Predictive	Prediction horizon	Strength	Limitation
Unidimensional metrics							
LOS/LWBS/Occupancy rate (5, 11, 14–16)	Mixed	Yes	Single variable	Reactive	None	Simple; widely available	Lacks predictive capacity
Multidimensional scores							
NEDOCS (17, 18)	ED	Partial (modified)	6 (i.e., number of ED patients, ED beds, hospital beds, ventilators in use in the ED; longest admission waiting time, waiting room time of the last patient called to a bed and number of patients admitted to the ED) (25)	Reactive	None	Quantitative threshold; multidimensional	Some variables not relevant in PEDs
EDWIN (19, 20)	ED	Tested	4 (i.e., number of staffed ED beds, attending physicians on duty; distribution of patients across triage categories and admission numbers) (20)	Reactive	None	Reflects workload and staffing balance	No pediatric adaptation
PEDOCS (21)	PED	Yes	6	Reactive	None	Tailored to PED; objective	Limited external validation
SOTU-PED (22)	PED	Yes	Perception-based	Reactive	None	Real-time feasibility	Subjective, center-specific
Predictive models							
Almeida model (23)	PED	Yes	Census, season, weather	Predictive	Daily	Simple and transferable	Single-site validation
SO-SAFED (24)	PED	Yes	Census, AI algorithms	Predictive	Several hours ahead	High accuracy; enables shift optimization	Requires technical infrastructure

While unidimensional metrics and multidimensional scores assess crowding retrospectively, predictive models reflect the transition toward anticipatory, data-driven management. Tools are included if they were originally developed for PEDs, adapted from adult-derived frameworks and subsequently evaluated in children, or if they introduced novel predictive approaches following the systematic review by Abudan et al. (1). The comparison summarizes each tool's origin, pediatric validation status, core variables, temporal function, and prediction horizon. NEDOCS and EDWIN represent benchmark adult-derived scores that have been evaluated in pediatric contexts, whereas PEDOCS and SOTU-PED are the only systems originally developed for pediatric emergency departments. Unidimensional metrics (e.g., length of stay, left without being seen, occupancy rate) remain the most frequently applied retrospective indicators in clinical practice. In contrast, the time-series forecasting model by Almeida et al. (23) and the AI-based SO-SAFED system (24) represent the first prospective predictive approaches in pediatric emergency care, illustrating the conceptual paradigm shift emphasized in this review. ED, adult emergency department; PED, pediatric emergency department; LOS, length of stay; LWBS, left without being seen.

Unidimensional crowding indicators such as length of stay (LOS), left without being seen (LWBS), and occupancy rates remain the most widely used metrics for PED crowding (5, 14). Their advantage lies in simplicity and data accessibility. However, they capture only one aspect of the complex crowding phenomenon. Metrics like LOS and LWBS were originally developed for adult EDs and require pediatric validation (15). Other studies have proposed additional operational markers such as waiting time, patient-to-nurse ratios, and daily patient volume (11, 16). While these are easy to quantify, they remain retrospective and cannot predict upcoming surges in demand.

Multidimensional scores originally developed for adult emergency departments, such as NEDOCS (17, 18) and EDWIN (19, 20), have been evaluated in pediatric settings. These studies confirmed correlations with established crowding metrics. These tools integrate several operational parameters, including patient volume, waiting time, and resource use, into a composite index. PEDOCS (21) and SOTU-PED (Score Objectif de Tension dans les services d'Urgences pédiatriques; Objective Tension Score in Pediatric Emergency Departments) (22) were the first scoring systems tailored specifically to PEDS. Both aim to provide objective, real-time identification of crowding levels and to account for the unique patient flow patterns in children. The modified NEDOCS (mNEDOCS) replaces adult-specific variables with pediatric-relevant indicators, such as the number of patients in resuscitation rooms (18). While this adaption improves contextual relevance in pediatric settings, validation remains limited by small cohorts and site-specific calibration.

The most recent and conceptually transformative development is the emergence of predictive modelling for PED overcrowding. In 2022, Almeida et al. (23) developed a time-series model to predict daily PED visits and support proactive resource planning. In addition to using a daily patient census as the core variable, the authors incorporated calendar effects (school vs. non-school days) and weather data. The model was based on data from over 600.000 admissions between 2010 and 2017 at a public hospital in Lisbon, Portugal. The objective was to identify temporal patterns and assess the influence of school-calendar and weather-related factors in order to develop a forecasting model for daily patient volumes. The results of the Almeida study revealed clear cyclical patterns in patient volumes. For example, a strong annual cycle was observed, with peaks in January and February, primarily driven by respiratory infections. Another cycle, occurring roughly every four months, correlated with school holiday periods. Using these cyclical patterns, the authors developed a time-series forecasting model with a mean absolute percentage error (MAPE) of 10.7% ± 1.1% in cross-validation, indicating promising predictive performance.

In 2025, Akbasli et al. (24) expanded on this concept with SO-SAFED (Shift Optimization and System for Anticipating and Forecasting Emergency Department Crowding), an artificial intelligence (AI)–based system using the daily patient census to predict overcrowding in a PED before it occurs. By calculating crowding several hours in advance, SO-SAFED allows proactive staff allocation and operational planning. The model was built around a single variable: the daily number of patient visits. Using data from over 350.000 PED visits, the authors developed and tested 20 time-series models (ranging from traditional statistical approaches to advanced deep learning algorithms) to forecast patient volumes. From this study, it became evident that modern AI models provide significantly better predictions than traditional statistical methods. A key advantage of AI-based approaches is automated model maintenance through MLOps (Machine Learning Operations) architectures. This allows the model to be continuously updated with current data, to respond to data drift, and to always select the best performing model for any given situation. During simulation, average forecast accuracy improved from 44% to 60%. These forecasts were then used to optimize physician staffing shifts increasing the total number of employed physicians. Schedules were adjusted in 69 out of 84 shifts, which led to a reduction of the number of patients per physician during peak hours by over 4, contributing to better care, more efficient use of resources and reduced workload. The results showed that with only one input, the daily patient volume, the system could effectively forecast crowding and guide real-time staffing adjustments. This approach can also be seen as unidimensional, but unlike the other unidimensional metrics, it was used to build a predictive model.

Both of these new models (23, 24) represent a paradigm shift from detection to prediction in pediatric emergency care settings. Unlike retrospective scores and metrics, they enable early intervention to potentially prevent the adverse downstream effects of overcrowding.

## 4 Discussion

Most existing PED overcrowding tools remain reactive, identifying problems only after capacity has been exceeded. Multidimensional scores such as NEDOCS and EDWIN offer structured, quantitative assessment but rely heavily on adult-derived variables (3, 13, 25). Their pediatric adaptations, including mNEDOCS and PEDOCS, demonstrate potential yet require multicenter validation to confirm generalizability. Retrospective unidimensional metrics continue to dominate daily operational monitoring due to simplicity. However, their limited scope prevents a nuanced understanding of complex system dynamics.

The emergence of predictive models marks a conceptual shift in how PED overcrowding can be understood and managed. Importantly, this paradigm shift is defined by temporal orientation rather than by the number of variables included. Predictive models—particularly those by Almeida et al. (23) and Akbasli et al. (24)—apply temporal modeling to historical data to generate forward-looking estimates of crowding. This also explains why predictive approaches may rely on a single primary input variable, such as patient census, while still representing a fundamentally different conceptual framework from retrospective unidimensional metrics. Their integration into clinical workflows could transform capacity management by enabling data-driven staffing and surge preparedness.

Nonetheless, predictive models face several important limitations. Current predictive models for pediatric emergency department overcrowding rely on historical utilization patterns and may therefore be vulnerable to structural disruptions such as pandemics, policy changes, seasonal surges, or shifts in care-seeking behavior. In addition, prospective evidence demonstrating clinical impact on patient outcomes, safety metrics, or quality of care is currently lacking. From a methodological perspective, AI-based time-series models carry inherent risks of overfitting and data leakage, particularly in single-center datasets. Few studies have compared predictive approaches with simpler baseline models in real-world deployment, including algorithm-driven staffing decisions. Ethical considerations surrounding algorithm transparency and data governance also warrant attention. Moreover, many existing neonatal machine-learning models lack explainability, a critical barrier to clinician trust and regulatory acceptance (26–28). Consequently, prospective, multicenter validation and evaluation of real-world clinical impact will be essential to establish robustness, safety, and added value beyond simpler baseline approaches.

In addition to these methodological challenges, several ethical and human factors remain insufficiently explored. Beyond technical validation, future research should address the ethical and operational consequences of predictive overcrowding management, including potential effects on triage fairness, staff workload, and patient experience. Clear governance structures, transparency of algorithmic decision support, and defined human oversight will be essential, as will the development of secure, interoperable data infrastructures to support multicenter implementation. Integrating these aspects will be crucial to ensure that future predictive tools not only enhance operational efficiency but also uphold ethical and human-centered standards of pediatric emergency care.

## 5 Conclusion

Approaches to assessing overcrowding in PEDs are evolving from retrospective metrics toward predictive, temporally oriented models. While unidimensional and multidimensional scores remain important for monitoring current system strain, predictive approaches introduce a forward-looking perspective that may enable anticipatory operational planning. However, current evidence is limited to single-center studies, underscoring the need for multicenter validation and robust governance frameworks before broader clinical implementation.
